# Emotion recognition and achievement prediction for foreign language learners under the background of network teaching

**DOI:** 10.3389/fpsyg.2022.1017570

**Published:** 2022-10-24

**Authors:** Yi Ding, Wenying Xing

**Affiliations:** School of Languages and Cultures, Shijiazhuang Tiedao University, Shijiazhuang, China

**Keywords:** emotion recognition, network teaching, achievement prediction, CNN-BiGRU, multimodal fusion

## Abstract

At present, there are so many learners in online classroom that teachers cannot master the learning situation of each student comprehensively and in real time. Therefore, this paper first constructs a multimodal emotion recognition (ER) model based on CNN-BiGRU. Through the feature extraction of video and voice information, combined with temporal attention mechanism, the attention distribution of each modal information at different times is calculated in real time. In addition, based on the recognition of learners’ emotions, a prediction model of learners’ achievement based on emotional state assessment is proposed. C4.5 algorithm is used to predict students’ academic achievement in the multi-polarized emotional state, and the relationship between confusion and academic achievement is further explored. The experimental results show that the proposed multi-scale self-attention layer and multi-modal fusion layer can improve the achievement of ER task; moreover, there is a strong correlation between students’ confusion and foreign language achievement. Finally, the model can accurately and continuously observe students’ learning emotion and state, which provides a new idea for the reform of education modernization.

## Introduction

The new generation of intelligent teaching system integrates artificial intelligence, learning analysis and personalized recommendation technology, which can not only promote the problem-solving ability of learners in autonomous environment, but also help to regulate learning emotion and enhance learning motivation. Learning emotion is an important part of learner modeling. More and more researchers pay attention to the effective detection of learning emotion and targeted intervention and guidance of negative learning emotion to improve teaching effect and teaching quality. Research shows that solving the confusion in learning in time can change the negative learning emotion into positive emotion, which is helpful to improve the learning achievement (Liu et al., 2018). However, the implicit learning confusion is strong, how to effectively detect it has become an important issue for current researchers. With the development of affective computing technology, effective detection and discovery of learning emotions will become a reality.

Among them, learners’ confusion in language learning is particularly prominent, which refers to the negative anxiety reaction produced by language learners in specific situations. Horwitz et al. (1986) believed that if all normal people have innate language acquisition mechanism, then acquired trigger input is very important, and the process of obtaining this input is closely related to personal learning motivation, anxiety, personality type, attitude, and other emotional factors. Emotional development includes the attention to students’ emotions and moods and the different effects of emotional changes on their studies. Emotion is a complex and transient internal reaction activated by external environment or internal stimulation (Gross, 2015). Positive emotions can bring positive and highly active subjective feelings to individuals, while negative emotions tend to bring negative and low active subjective feelings to individuals.

Achievement prediction is one of the most important research issues in the field of educational data mining, which is a hot issue that many researchers at home and abroad pay attention to. Through the prediction of student achievement, it can provide timely warning information for educators. Kriegel et al. (2007) pointed out that the application prospect of machine learning method in the field of education is very wide, and the data modeling of learning achievement can be carried out by using this technology to realize the prediction of learning achievement (Kriegel et al., 2007). However, the current prediction modeling of academic achievement mainly focuses on the construction of the achievement prediction model, the analysis of the prediction model and the evaluation of the prediction model. In fact, emotional state has a great impact on learners’ academic achievement, such as the widespread confusion in the learning process. If the learners are in a state of confusion for a long time, they will have a sense of frustration, which is not conducive to the effective learning of knowledge. Persistent confusion will lead to the decline of learners’ interest in learning and the lack of learning motivation, which will affect their academic achievement. Especially for online learning, the more confused the learners are about the course content, the lower the retention rate of the course, and thus the completion rate of the course is affected (Baker et al., 2012; Vail et al., 2016).

The purpose of this paper is to apply the ER of foreign language learners in the process of language learning to the construction of online learning environment, so as to improve the learner model, provide technical support for the realization of emotional interaction, and mine learning behavior. Therefore, this paper constructs a multi-modal ER model based on CNN-BiGRU, and puts forward a achievement prediction model based on emotional state assessment, so as to further explore the correlation between confused emotion and academic achievement.

## Related works

### Confusion in foreign language learning

Linguists have studied language learning anxiety from different perspectives. For example, Horwitz et al. (1986) pointed out that foreign language anxiety is related to language learning in classroom, and is generated in the process of language learning. It is a unique and complex self-awareness, belief, emotion and behavior in language learning, including communication fear, test anxiety and fear of negative evaluation; Rajitha and Alamelu (2020) pointed out that language anxiety is the main factor affecting the affective factors of second language learners, and the factors causing language anxiety include various types of examination results, oral narration, written expression, self-confidence and self-esteem in the process of language learning (Rajitha and Alamelu, 2020); The interdisciplinary theories and methods introduced by Dewaele (2005) can promote the gradual development of emotion research in second language acquisition; Bielak and Mystkowska-Wiertelak (2020) used the situational experiment method to study the use of emotion regulation strategies in English learning of Polish college students.

The research mainly focuses on the influence of positive or negative emotions on English learning and introduces the appropriate adjustment measures. Han and Xu (2020) adopted emotion-oriented, assessment-oriented and situational-oriented adjustment strategies for different students’ emotions, and studied the emotional changes of second language learners in the writing process and the adjustment strategy scheme. Li (2020) pointed out that the study of language learners’ emotion in learning is an essential teaching practice in the study of foreign language learning psychology. Wang (2014) carried out cognitive reconstruction on 32 undergraduate students under the guidance of rational emotional behavior therapy with the help of psychological outpatient technology for the treatment of general social anxiety. The results show that rational emotional behavior therapy is helpful to reduce learners’ oral English anxiety.

### ER of learners

Different researchers pay attention to different models of learning emotion, and the focus is also different. Wu et al. (2008) applied learning emotion to distance learning system, and proposed a new learning emotion modeling method based on OCC emotion model and two-dimensional emotion model, to realize the interaction between cognition and emotion in Distance Teaching. In order to solve the shortcomings of traditional learning model (Wang and Gong, 2011) introduced learning style and learning emotion and other factors to construct a perfect e-learning student model to solve the emotional lack of online teaching system, and improve the intelligence and personalized role of the system. Shi et al. (2007) designed experiments to collect biological signals such as skin conductivity, blood pressure and brain waves to construct a circular emotion model. They found that participation and confusion are the two most frequent emotions in e-learning learning activities, which improve the learning effect in e-learning.

The emotion modeling of learners needs machine learning model to model the multimodal data such as picture physiology and text collected by researchers, so as to realize the recognition and discovery of learning emotion of complex data. Kapoor et al. (2007) established an emotion model based on the collected facial expression images and heart rate data, and realized the detection of learning emotion based on Dynamic Bayesian network. With the improvement of data processing technology, researchers can obtain better ER results by processing and analyzing multimodal data, and improve the accuracy of Emotion Modeling (Ling et al., 2021). In addition, the data of learning emotion come from various learning scenes, including facial expression pictures, physiological data and text data. Ekman and Lavoué (2017) developed a set of emotion coding system based on facial action features. The system can identify six emotions, including happiness and surprise, by encoding facial features according to the achievement differences of different faces. This study provides important inspiration for Learning Emotion Modeling with facial expression pictures (Ekman and Lavoué, 2017). Jin et al. (2016) constructed a learning emotion measurement model, including user data module, analysis and diagnosis module, emotion integration module and feedback module, aiming to solve the problem of lack of emotional communication in online learning.

The emerging deep learning methods in recent years have well made up for the defects of the two methods based on machine learning and sentiment dictionary. Yin and Schütze (2016) used multi-channel convolutional neural networks of different sizes for sentence classification. Chen et al. (2018) proposed a multi-channel convolutional neural network model, which used multiple convolutional neural networks to extract the multi-faceted features of sentences, and achieved good results in the sentiment analysis task of Chinese microblog. However, CNN-based sentiment classification has the problem that it cannot consider the semantic information of sentence context. Alayba et al. (2018) proposed a combination of CNN and LSTM model for Arabic sentiment analysis and achieved good classification results. Zhang et al. (2018) used the Convolution-GRU model to discriminate the sentiment polarity of Twitter hate comment text. Yuan et al. (2019) proposed a sentiment analysis model based on multi-channel convolution and bidirectional GRU network, and introduced an attention mechanism on BiGRU network to automatically pay attention to features with strong influence on sentiment polarity. In view of the excellent performance of the neural network model integrating attention mechanism, this paper also introduces attention mechanism in the task of text sentiment orientation analysis, so that the network model can pay more attention to the words that contribute a lot to the text sentiment polarity.

### Prediction of learners’ achievement

In this paper, we propose a learning model based on Bayesian neural network and Bayesian regression model. Yuan and Zhu (2021) based on the data of various factors of students’ English learning, the researchers used random forest algorithm to model scores and predict the passing rate of CET-4. Wu (2017) collected data on Online Learners’ demographic information, autonomous learning behavior and writing learning behavior, and constructed multiple learning achievement prediction models using decision tree, support vector machine, neural network, Bayesian network and other machine learning algorithms. Such models can provide advance organizers and strengthen learning discussion supervision to promote effective teaching strategies (Wu, 2017). Sun et al. (2016) used k-means algorithm to cluster students’ degree English scores, determined a more specific score distribution interval, and used C5.0 classification algorithm of decision tree to carry out achievement prediction modeling and analysis, so as to realize the prediction model of students’ degree application achievement. Through this model, the coping strategies between undergraduate students’ English learning level and adult English test scores were proposed, which helps to improve the learning effect (Sun et al., 2016).

Although studies have shown that there is a strong correlation between learners’ emotions and academic achievement, few scholars have focused on exploring the internal relationship between them. Research made a prediction based on learning behavior, while the prediction based on learning emotion is still less. Therefore, the purpose of this study is to explore the internal relationship between learning confusion and academic achievement, so as to uncover the quantitative model relationship between confusion and correct test questions, and to establish relevant prediction models.

## Multimodal ER based on CNN-BIGRU

### Overall structure

**Figure 1:**
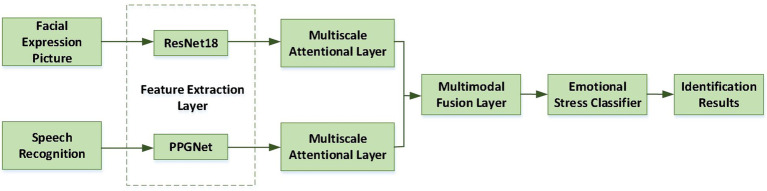
Multimodal ER framework.

In this paper, a tensor fusion method based on low rank decomposition is introduced. This method can focus on the information inside and between modes, and aggregate the interaction between modes, so that the features of facial expression and pulse signal are more complementary. The framework of MA-TFNet is shown in Figure 1.

Firstly, the high-dimensional features of facial expressions and speech signals are extracted, and the high-dimensional features are input into Bi-GRU network for training. Then, using the output of Bi-GRU network of two modes, the attention distribution of each mode at each time is calculated. The feature vectors of two modes with attention weight are input into the multimodal feature fusion module, and the fused eigenvectors are used as the input of the fully connected network. After training, the data to be identified is input into the network to get the output of ER.

### Feature extraction

**Figure 2:**
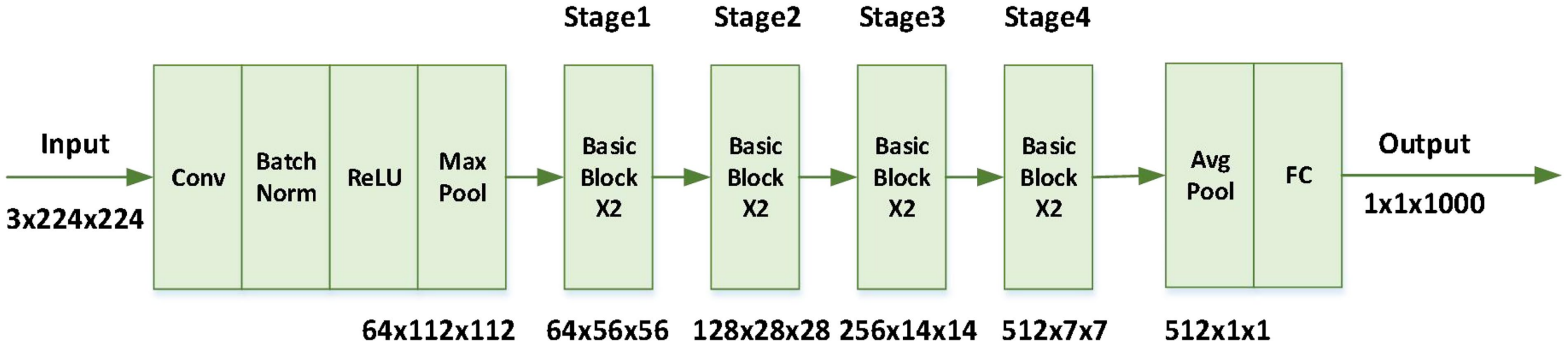
Network structure of resnet18.

For feature extraction of facial expression image sequence, res net18 network is used in the model. ResNet based on residual structure has a strong ability of feature extraction. In computer vision tasks, it is often used as a basic convolution neural network to extract image features. Moreover, it has a strong generalization ability and can perform well in different data sets. At present, ResNet has been widely used in various image-related tasks, where, as shown in Figure 2, BasicBlock is a residual structure formed by two 3 × 3 convolution.

**Figure 3:**
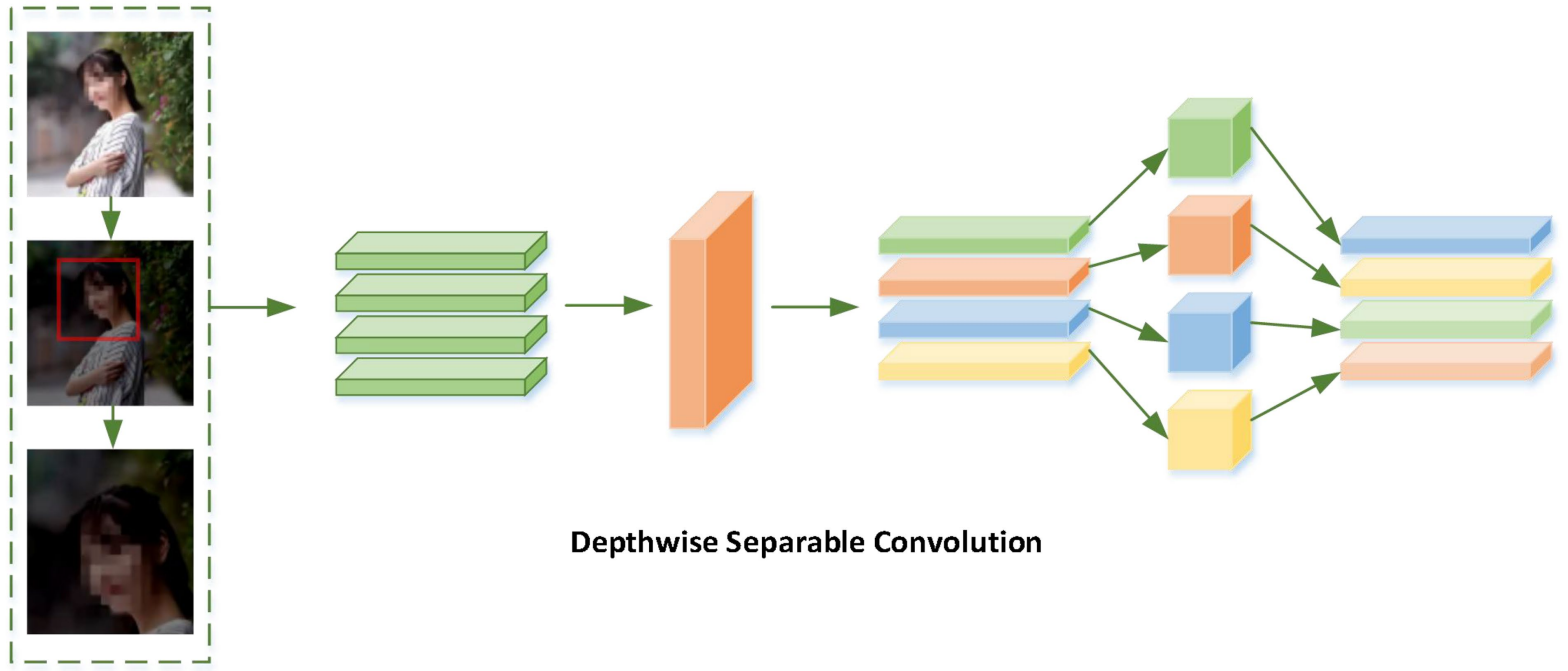
Bi-GRU process.

The basic idea of Bi-GRU is to set two different data flow directions for input data, forward propagation and backward propagation, which connect the same output layer. Through this structure, the network can not only pay attention to the information of the past time of the input data, but also focus on the information of the future time of the input data. Bi-GRU focuses on the information of past time and future time of input data by setting forward calculation and backward calculation, where the order of information flow in the forward calculation layer is from the past to the future, receiving the input of the current time and the output of the hidden layer at the previous time; while the order of information flow in backward calculation layer is from the future to the past, receiving the input of the current time and the hidden layer of the next time. Finally, the output layer outputs two hidden layer States, one from the forward computing layer and the other from the backward computing layer, as shown in Figure 3.

The image features $f=\left\{f_{1},f_{2},f_{3},\ldots ,f_{t}\right\}$ is input into Bi-GRU network, and Forward( ·) is defined as the Forward computation function of the network, while Backward( ·) is the Backward computation function of the network, as shown in Equations (1), (2):


(1) $${\vec{h}}_{t}=\mathrm{Forward}\left(f_{t},{\vec{h}}_{\left(t-1\right)}\right)$$

(2) $${\overset{\leftarrow }{h}}_{t}=\mathrm{Backward}\left(f_{t},{\overset{\leftarrow }{h}}_{\left(t+1\right)}\right)$$

Among them, the ${\vec{h}}_{t}$ is network layer before *t* time to calculate the output, $\overset{\leftarrow }{h}$ is the network layer to calculate the output after *t* time. ${\vec{h}}_{\left(t-1\right)}$ and ${\overset{\leftarrow }{h}}_{\left(t+1\right)}$ represent the network in *t*−1 h after the forward calculation of the output layer and to calculate the output layer. When the timing information of the high-dimensional features of the input data is fully learned by the network, the state information of the network is output as follows:


(3) $$H=\left[\left[{\overset{\leftarrow }{h}}_{1},{\vec{h}}_{1}\right],\left[\overset{\leftarrow }{h_{2}},{\vec{h}}_{2}\right],\ldots ,\left[\overset{\leftarrow }{h_{t}},{\vec{h}}_{t}\right]\right]$$

Define the input of facial expression Bi-GRU subnet as $x_{{\mathrm{face}}_{1}},x_{{\mathrm{face}}_{2}},\ldots ,x_{{\mathrm{face}}_{t}}$ .

Then the output of the hidden layer of Bi-GRU network is:


(4) $$H=\left[\left[{\overset{\leftarrow }{h}}_{1},{\vec{h}}_{1}\right],\left[{\overset{\leftarrow }{h}}_{2},{\vec{h}}_{2}\right],\ldots ,\left[{\overset{\leftarrow }{h}}_{t},{\vec{h}}_{t}\right]\right]$$

Therefore, the hidden layer output of facial expression Bi-GRU is:


(5) $$H_{i}=\left[\left[{\overset{\leftarrow }{h}}_{\mathrm{face}{\;}_{1}},{\vec{h}}_{{\mathrm{face}}_{1}}\right],\left[h_{{\mathrm{face}}_{2}},{\vec{h}}_{{\mathrm{face}}_{2}}\right],\ldots ,\left[{\overset{\leftarrow }{h}}_{\mathrm{face}{\;}_{t}},{\vec{h}}_{{\mathrm{face}}_{t}}\right]\right]$$

The hidden layer output of speech signal Bi-GRU is:


(6) $$H_{p}=\left[\left[{\overset{\leftarrow }{h}}_{ppg_{1}},{\vec{h}}_{ppg_{1}}\right],\left[{\overset{\leftarrow }{h}}_{ppg_{2}},{\vec{h}}_{ppg_{2}}\right],\ldots ,\left[{\overset{\leftarrow }{h}}_{ppg_{t}},{\vec{h}}_{ppg_{t}}\right]\right]$$

When designing the temporal attention mechanism, we hope to ensure that the unique information of each mode will not be lost, and at the same time, the information of the other mode can be combined. Therefore, this paper calculates the input information of the two modes according to different proportions. At the same time, the proposed attention mechanism can combine the context information of temporal information and refer to the practice of ECA-Net in the network structure design, where the input of the two modal information will pass through the global average pooling layer and the one-dimensional convolution layer, and then calculate the attention weight.

The specific attention calculation methods are as follows:

Input ${\vec{h}}_{face}\in {\mathbb{R}}^{T\times d},{\overset{\leftarrow }{h}}_{face}\in {\mathbb{R}}^{T\times d}$ , which, respectively, represent the two facial expressions of bidirectional GRU order and reverse order.

Input ${\vec{h}}_{ppg}\in {\mathbb{R}}^{T\times d},{\overset{\leftarrow }{h}}_{ppg}\in {\mathbb{R}}^{T\times d}$ , which are the hidden vectors of speech signals.

### Feature fusion

For the j -th recognition classification, the new classification probability $q_{j}\left(x\right)$ obtained after processing according to the Rule can be expressed as follows:


(7) $$q_{j}\left(x\right)=q_{j}^{\prime }\left(x\right)/\overset{m}{\underset{j}{\sum }}q_{j}^{\prime }\left(x\right)$$

Where the calculation equation of $q_{j}^{\prime }\left(x\right)$ is as follows:


(8) $$q_{j}^{\prime }\left(x\right)=\mathrm{rule}\left(p_{ij}\left(x\right)\right)$$

Finally, the classification label is obtained as follows:


(9) $$w\left(x\right)=\mathrm{argmax}\left(q_{j}\left(x\right)\right)$$

The classification corresponding to the largest value is the final classification result. That is, $q_{j}^{\prime }\left(x\right)$ can be expressed as


(10) $$q_{j}^{\prime }\left(x\right)=\overset{n}{\underset{i=1}{\sum }}p_{ij}\left(x\right)$$

## Learners’ achievement prediction model based on emotion state assessment

### Multi polarization emotional state assessment

Based on the vectorized representation of foreign language learners’ multi-polarized emotion, the multi-polarized emotion vector of foreign language learners in each class hour t can be calculated, and the multi-polarization emotion state matrix can be constructed to evaluate the change characteristics of their multi polar emotional state. On this basis, it can further analyze the phased dominant emotion of learners in class hour t, so as to assess the emotion tendency of learners, and then provide targeted personalized teaching intervention for learners with different emotion tendencies.

Step 1: Calculate the multi-polarization emotion state matrix of learners, as shown in Equation (11)


(11) $$\mathrm{Mat}\left(L_{i}\right)=\left[\begin{array}{ccc} v_{1,1} & \ldots & v_{1,\mathrm{utn}}\\ \ldots & v_{i,t} & \ldots \\ v_{8,1} & \ldots & v_{8,\mathrm{utn}} \end{array}\right]$$

Where $\mathrm{Mat}\left(L_{i}\right)$ represents the i -th learner’s multi-polarization emotion state matrix; utn represents the current class progress, and u tn $\in Z^{+}$ ; $v_{i,t}$ represents the emotional intensity value of the i -th emotion in t period, and $v_{i,t}\in v_{t}$ .

Step 2: Calculate the dominant emotion in stages. Based on the multi polar emotion vector analysis of learners, it can be found that learners usually show a single polarity emotion type at the same time. Therefore, through the vectorization of learners’ periodic multi polarization emotions, we can further analyze the learners’ phased dominant emotions $Dmt_{pst}\left(v_{t}\right)$ . When the intensity value of a certain emotion is the largest element in $v_{t}$ , the phased dominant emotion of the learner $Dmt_{pst}\left(v_{t}\right)$ in class period t is defined, as shown in Equation (12):


(12) $$Dxt_{pst}\left(v_{t}\right)=\max\left(v_{t}\right)$$

Where $Dmt_{pst}\left(v_{t}\right)$ represents the key value pair of emotional polarity and intensity of the first learner’s dominant emotion in t period.

Step 3: Construct a multi-polar emotional state change chain. Based on the phased dominant emotion calculation, the multi-polar emotion states of each course stage can be obtained, and thus the multi-polar emotion state change chain can be constructed, as shown in Equation (13):


(13) $$\begin{array}{l} Dmt_{pst}-\mathrm{chain}\\ =\left[Dmt_{pst}\left(v_{1}\right),\;Dmt_{pst}\left(v_{2}\right),\ldots ,\;Dmt_{pst}\left(v_{t}\right),\ldots ,\;Dmt_{pst}\left(v_{utn}\right)\right] \end{array}$$

Where $Dmt_{pst}-$ chain is the multi-polar affective state change chain of the i -th learner in the current course progress. $Dmt_{pst}\left(v_{t}\right)$ is the key pair of emotion polarity and intensity.

Step 4: Identify the emotional tendency of learners. The traditional method to judge the user’s emotional orientation is based on the positive and negative of the calculation results by means of superposition calculation of the user’s emotional intensity. However, the limitation of this method is that local extreme emotions can easily counteract or even cover the global dominant emotions, which leads to the low classification accuracy of the method. Therefore, this paper counts the frequency of each polarity’s phased dominant emotion in the course progress, and the most frequent emotional polarity is the learners’ emotional inclination, as shown in Equation (14).


(14) $${\mathrm{Sent}}_{\mathrm{tend}}\left(L_{i}\right)=m\left(Dmt_{pst,p}-\mathrm{freq}\;\right)$$

Where ${\mathrm{Sent}}_{\mathrm{tend}}\left(L_{i}\right)$ represents the i -th learner’s emotional tendency; $Dmt_{pst,p}-$ freq represents the frequency that the emotion with polarity of p dominates the stage in the current class schedule.

### Achievement prediction

Through emotional feature selection, this paper uses C4.5 algorithm to predict student assembly. The algorithm selects features based on information gain rate, and uses the maximum information gain rate as the branch standard of decision-making features until all subsets have the same class of data. Let the sample training set *D* have d emotion state data and m different categories, the m different categories are defined as $L_{i}\left(i=1,2,\cdots ,m\right).$ Let $d_{i}$ be the number of samples in class $L_{i}$ , and the expected information amount of emotion state data set D is:


(15) $$I\left(D\right)=I\left(d_{1},\ldots ,d_{m}\right)=-\overset{m}{\underset{i=1}{\sum }}P_{i}\times \log_{2}\left(P_{i}\right)$$

Where $P_{i}$ represents the probability that the sample belongs to class $L_{i}$ , that is, $P_{i}=\frac{d_{i}}{d}$ .

Set emotion features H has n different discrete values $\left\{h_{1},h_{2},h_{3},\cdots ,h_{n}\right\}$ , the data set D is divided into n subset $\left\{D_{1},D_{2},D_{3},\cdots D_{n}\right\}$ . Let $d_{ij}$ be the number of samples belonging to $L_{i}$ class in subset $D_{j}$ . Then, the entropy of *H* partition sample subset is:


(16) $$E\left(H\right)=\overset{n}{\underset{j=1}{\sum }}\frac{\left|D_{j}\right|}{d}I\left(d_{1j},\ldots ,d_{mj}\right)$$

The expected information of subset $D_{j}$ is:


(17) $$I\left(d_{1j},\ldots ,d_{mj}\right)=\overset{m}{\underset{i=1}{\sum }}P_{ij}\times \log_{2}\left(P_{ij}\right)$$

Where $P_{ij}=\frac{d_{ij}}{\left|D_{j}\right|}$ is the probability that each data sample in subset $D_{j}$ belongs to category $L_{i}$ .

Therefore, the information gain of H as a branch node for sample training set partitioning is:


(18) G(H)=I(D)−E(H)

## Experiment and analysis

### Data set

The selection of data set is very important for ER. Because seven categories of ER is used, THCHS30 data set is selected as sound data set,^1^ which is an open Chinese speech data set that released by the Center of Speech and Language Technology (CSLT) of Tsinghua University. For video data set, FER2013 Kaggle Challenge data set is used.^2^ The face data is composed of 48×48 pixels, and each picture has a corresponding label, a total of seven expressions, numbered from 0 to 6. The original database has 73,500 images of learners’ facial expressions, each image has a corresponding label, a total of seven expressions, numbered from 0 to 6. At the same time, the platform automatically annotates the emotion type and intensity of each image. For example, in 0001_02_03_0004, 0001 represents the subject number, 02 represents the emotion type, 03 represents the emotion intensity, and 0004 represents the image number.

### Results and discussion

#### Model validation

**Figure 4:**
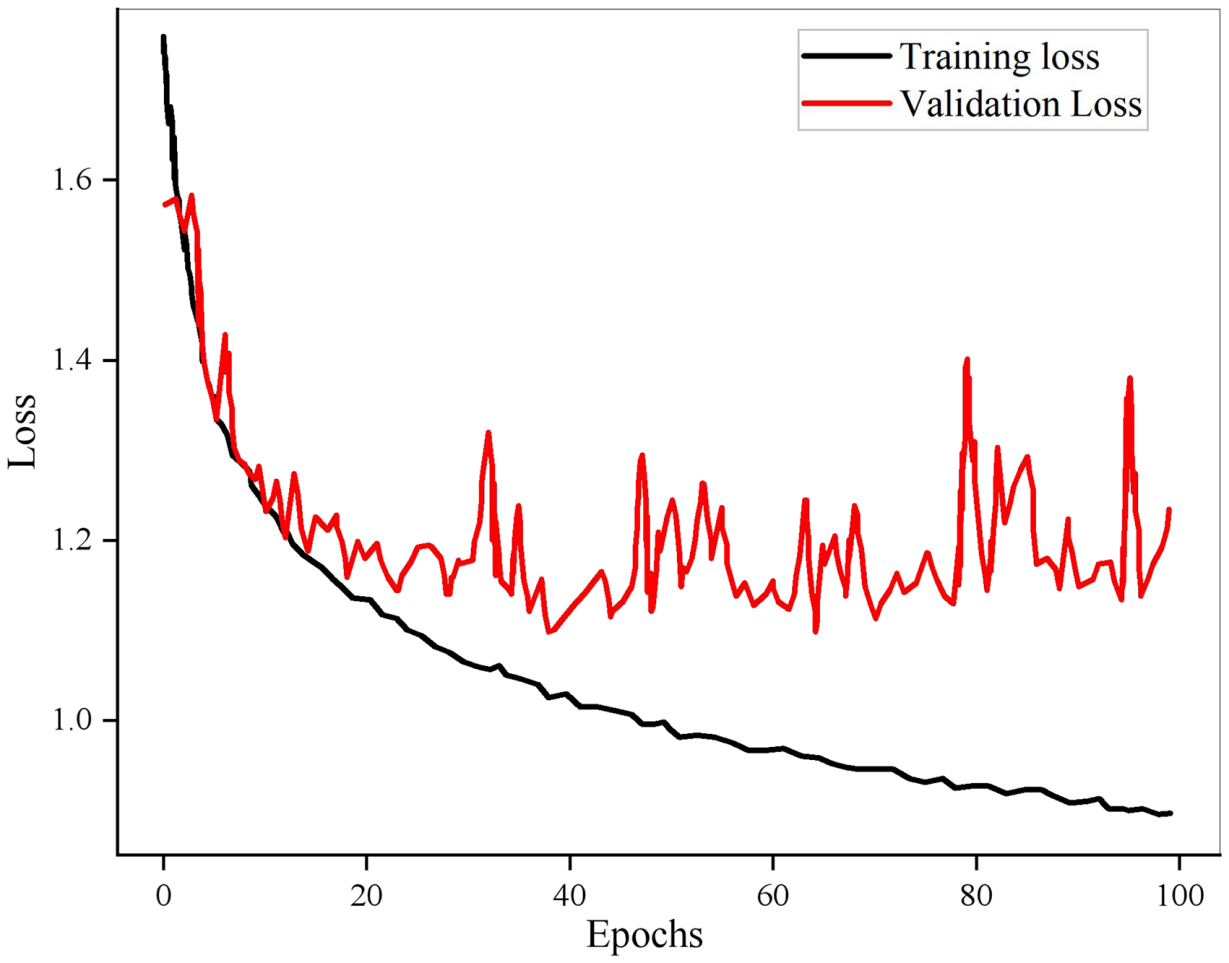
Results of model training.

The loss function of the model changes with the number of iterations as shown in Figure 4.

It can be found from the figure that the accuracy of the test set is not much different from that of the training set, and the loss value of the test set is also consistent with the loss value of the training set, indicating that the model can be applied in practice. Bi-GRU structure enables the network to access the information of past time and future time in multimodal data at the same time, capture the change of emotional stress, and fully learn the information.

#### ER results

**Figure 5:**
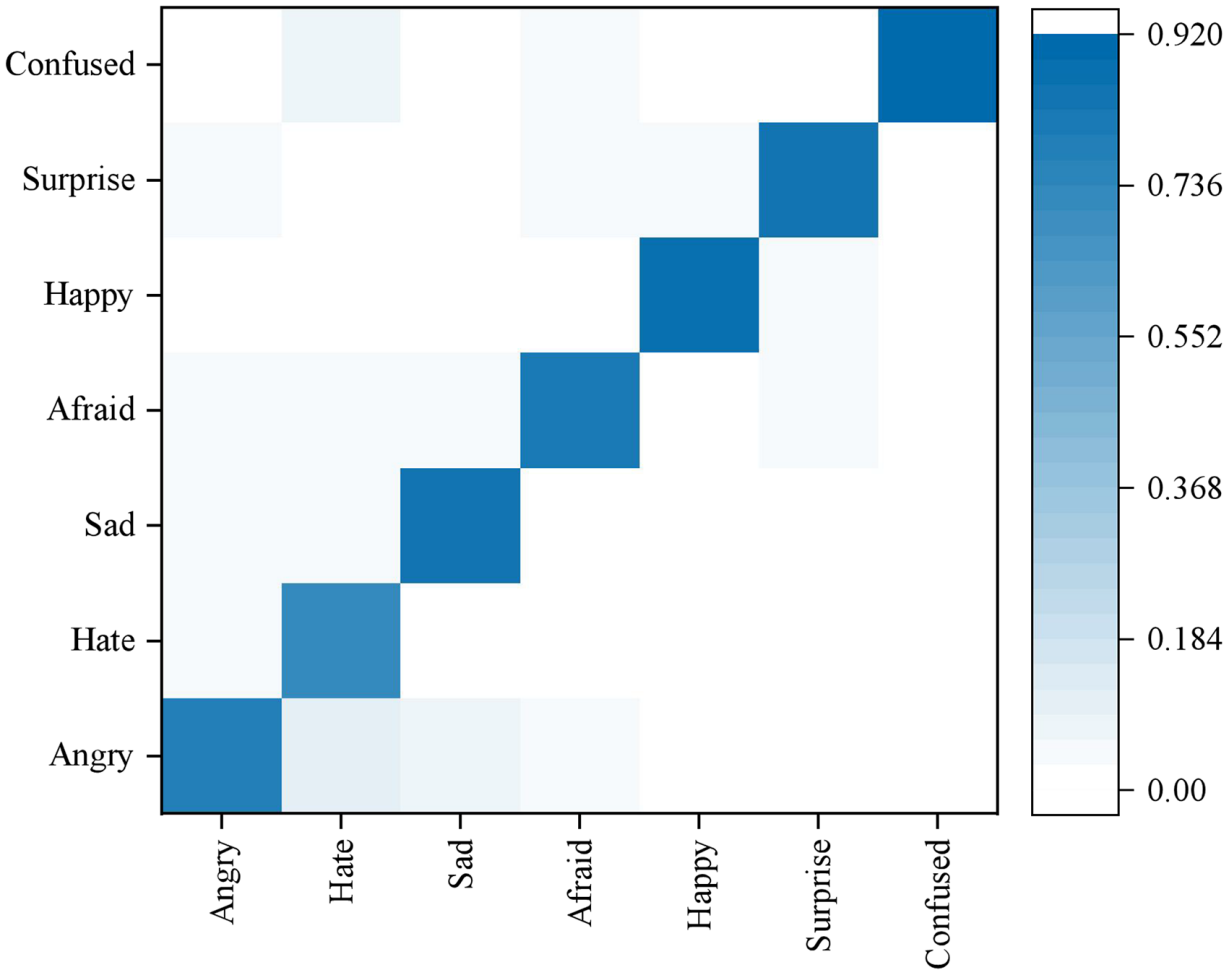
Confusion matrix of different ER.

The confusion matrix of ER results based on the fusion of decision level is shown in Figure 5. Its accuracy rate is higher than that of ER based on single mode such as voice or facial expression.28708 test charts were selected from the FER2013 dataset, and the training set of THCHS30 data set was trained. The results showed that the average recognition rate was 82.1%. Compared with the single-mode ER results of speech and image, it is found that the accuracy of the multi-modal ER results after fusion has been greatly improved, which shows that the algorithm can be applied to classroom teaching. At the same time, in foreign language class, learners’ confused emotions are widely distributed and need to be focused on.

In addition, when multi-scale attention layer and multi-modal fusion layer are included, the accuracy rate of learners’ ER is high, which shows that the self-attention layer and multi-modal fusion layer proposed in this paper can improve the achievement of multimodal ER task by superposition, proving the effectiveness of the method.

#### Learners’ emotional changes based on time characteristics

**Figure 6:**
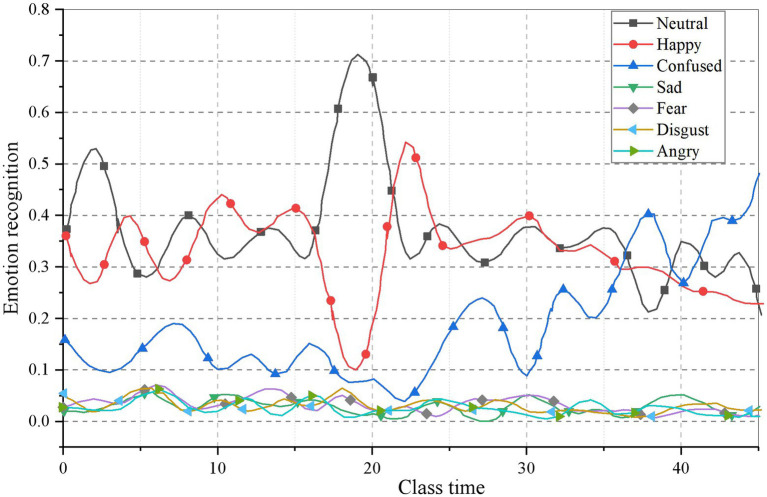
Results of students’ ER in the first week.

**Figure 7:**
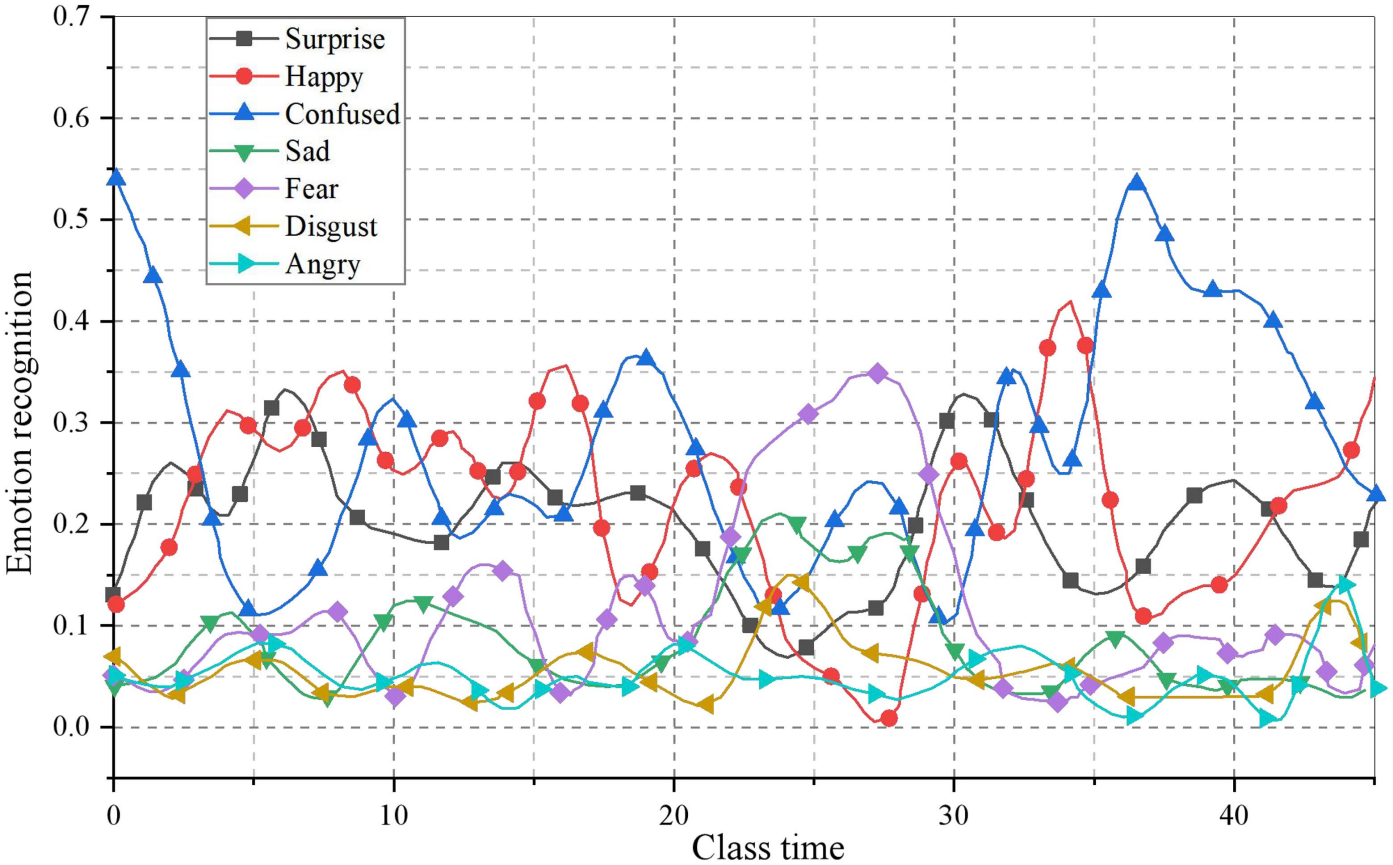
Results of students’ ER in the sixth week.

This paper analyzes the video data of Multimedia English teaching class in a university in Xi’an. After 6 weeks of teaching application, students’ emotions are collected, as shown in Figures 6, 7.

It can be clearly seen from the figure that students’ emotions are different in each cycle. In the first week, students’ learning emotions are mainly natural. Combined with the actual situation of teaching, it is found that the basic structure of grammar teaching is the content of this class, and teachers mainly teach. While in the sixth week, the teachers found that the students’ enthusiasm for learning was greatly stimulated by the interaction between the teachers and the students in the classroom.

#### Correlation between learners’ emotional state and achievement

**Figure 8:**
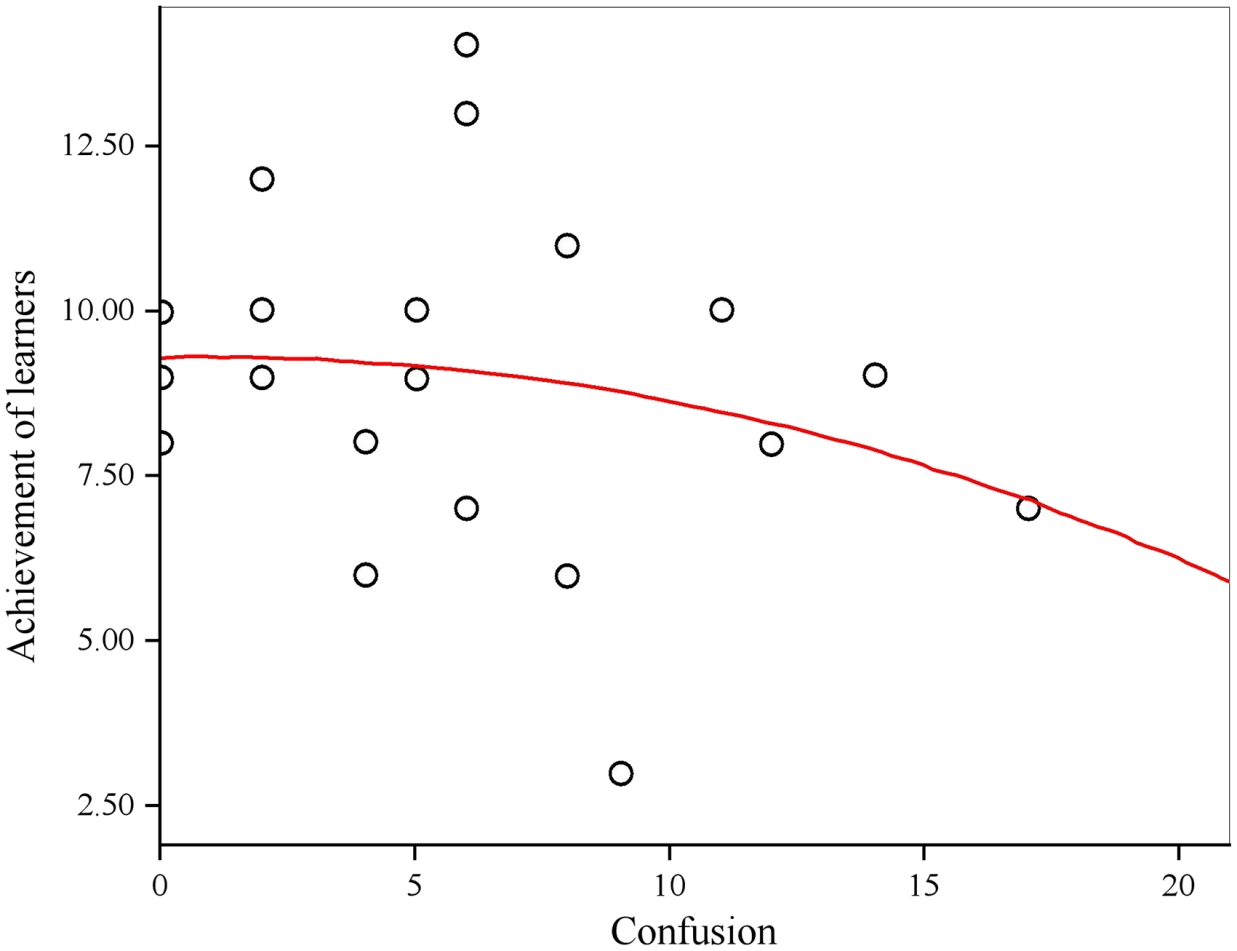
Fitting curve between learners’ confusion and achievement.

As mentioned above, language learners’ confusion is particularly prominent, which can bring low active subjective feelings to learners. The section “ER results” also confirm this view. Therefore, this study uses two variable indicators, namely learning confusion and view resolution, to predict the right and wrong of the test questions of learners, so as to achieve the purpose of predicting achievement. Self-report is often used to define emotional labels in learning emotion detection, where students can determine whether their emotional state is in a state of confusion according to the options of self-report. It can be seen from Figure 8 that the quadratic curve equation fitted by linear regression can better reflect the relationship between learners’ confused emotion and achievement.

When the number of confused questions was more, the score also decreased significantly. From this we can draw the following conclusions: learning confusion has a more obvious impact on learning achievement, too much learning confusion will lead to the increase in the number of errors, reduce the accuracy rate, and lead to the decline of the overall score; The learners who are puzzled by the increasing number of test questions may not grasp the whole knowledge firmly enough, which leads to greater difficulty in choosing almost all knowledge points, thus affecting the whole judgment. Therefore, if we can help learners to solve the current confusion in a timely manner, it may help learners to have a new understanding of learning, so as to improve their academic achievement.

Learning behavior has a great impact on learning achievement. Learning behavior indirectly reflects the level of learners’ emotional state. If some behaviors can be seen that learners are in a relatively negative emotional state, they will further affect their academic achievement. Learning confusion is positively related to the change of students’ achievement.

### Application scenarios

**Figure 9:**
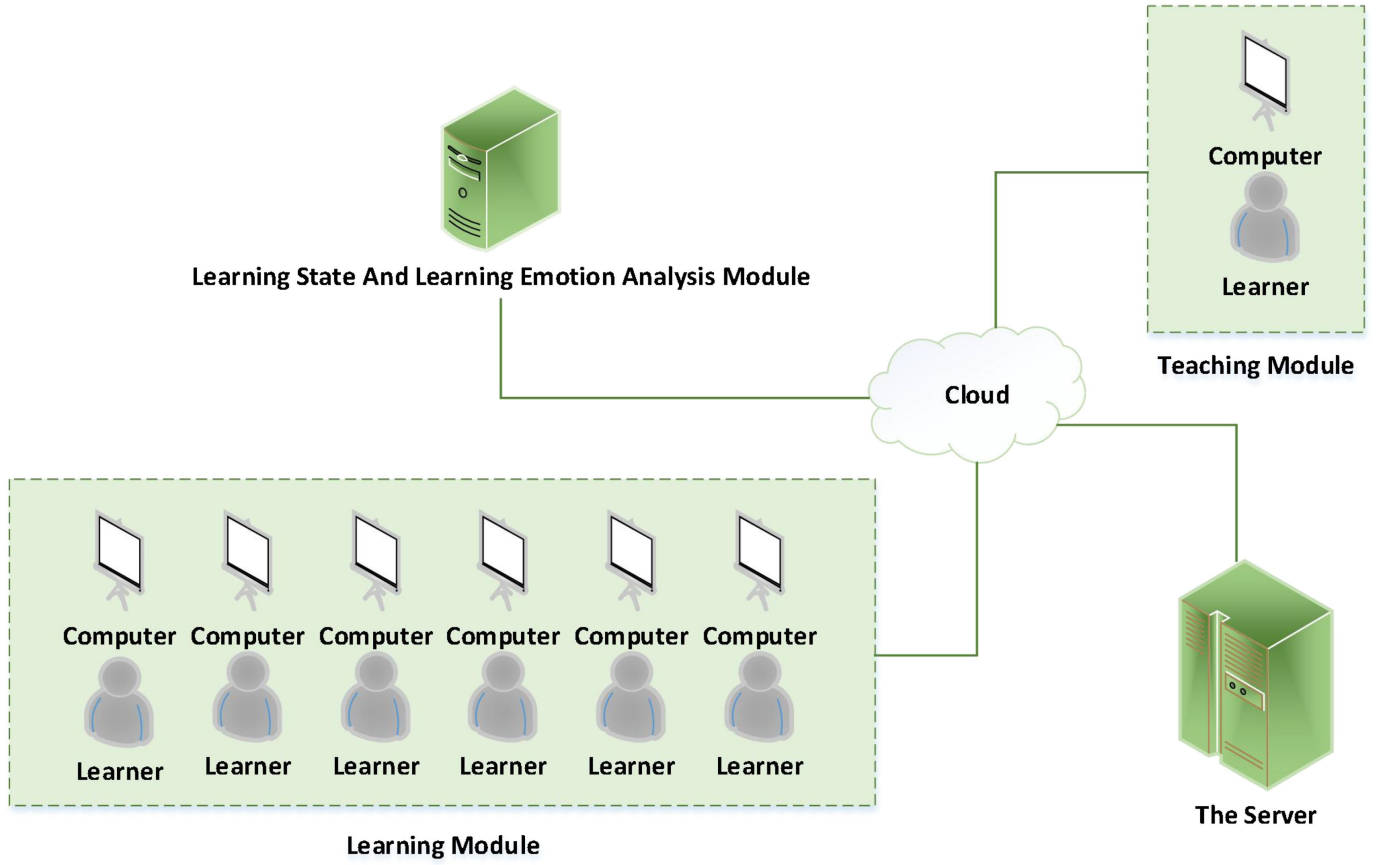
Deployment of emotion analysis module in network teaching system.

The experimental results show that the multimodal foreign language learners’ ER model proposed in this paper can accurately and continuously observe students’ learning emotions and states. Therefore, the analysis module of learning state and learning emotion can be added to the existing network teaching system of colleges and universities. As shown in Figure 9, the deployed system consists of learning module, teaching module, learning state and learning emotion analysis module, and server module. These four parts exchange data through the cloud to ensure the normal operation of the network teaching system.

The system starts to implement after the course starts. It collects the learners’ learning state and learning emotion information according to the time period, and records the collected effective data. Then the data are preprocessed and analyzed by the algorithm to get the feedback result. Finally, according to the feedback results, data analysis is carried out to make a positive impact on learners.

## Conclusion

This paper constructs a multi-modal ER model based on CNN-BiGRU, and proposes an achievement prediction model based on emotional state assessment to further explore the relationship between confused emotion and academic achievement. Moreover, it analyzes the influence of learners’ emotional state on learning process from different levels. The results show that in the learning of foreign language, learners’ confusion is widely distributed and needs to be focused on; while too much confusion will lead to more mistakes and lower correct rate. In the characteristics of time and space, the change of teaching methods can affect students’ emotional changes. Finally, in the future, the model can be integrated into the existing network teaching system of colleges and universities to accurately and continuously observe students’ learning emotions and states, and then help teachers adjust teaching strategies in time.

## Data availability statement

The original contributions presented in the study are included in the article/supplementary material, further inquiries can be directed to the corresponding author.

## Ethics statement

This study were reviewed and approved by School of Languages and Cultures, Shijiazhuang Tiedao University. The participants provided their written informed consent to participate in the study.

## Author contributions

YD was responsible for the conception of research ideas. WX was responsible for data collection. All authors contributed to the article and approved the submitted version.

## Funding

The study was supported by “Teaching reform project of Hebei Provincial Department of Education: Research on interdisciplinary teaching of small languages in engineering colleges and universities under the background of the “Belt and Road,” China (grant no. 2020GJJG175), Hebei Provincial Science and technology department in 2022: Research on the promotion strategy of national cultural soft power from the perspective of Chinese internationalization process, China, Project of Hebei Provincial Federation of Social Sciences, Research topic: Research on Hebei Province’s integration into the “Belt and Road” construction and development strategy: Taking Sino-Russian science, education and cultural production capacity cooperation as an example, Project category: Youth project, China (grant no. 20200303118), and Department of Science and Technology of Hebei Province in 2022: Research on the strategy of promoting national cultural soft power from the perspective of Chinese internationalization.

## Conflict of interest

The authors declare that the research was conducted in the absence of any commercial or financial relationships that could be construed as a potential conflict of interest.

## Publisher’s note

All claims expressed in this article are solely those of the authors and do not necessarily represent those of their affiliated organizations, or those of the publisher, the editors and the reviewers. Any product that may be evaluated in this article, or claim that may be made by its manufacturer, is not guaranteed or endorsed by the publisher.

## Footnotes

^1^ http://www.openslr.org/18/

^2^ https://www.kaggle.com/
